# Tractography-based priors for dynamic causal models

**DOI:** 10.1016/j.neuroimage.2009.05.096

**Published:** 2009-10-01

**Authors:** Klaas Enno Stephan, Marc Tittgemeyer, Thomas R. Knösche, Rosalyn J. Moran, Karl J. Friston

**Affiliations:** a Laboratory for Social and Neural Systems Research, Institute for Empirical Research in Economics, University of Zurich, Switzerland; b Wellcome Trust Centre for Neuroimaging, Institute of Neurology, University College London, 12 Queen Square, WC1N 3BG, London, UK; c Max Planck Institute for Neurological Research, Cologne, Germany; d Max Planck Institute for Human Cognitive and Brain Sciences, Leipzig, Germany

**Keywords:** Anatomical connectivity, Effective connectivity, Dynamic causal modelling, DCM, Bayesian model selection, Model evidence, Bayes factor, Probabilistic tractography, Diffusion weighted imaging

## Abstract

Functional integration in the brain rests on anatomical connectivity (the presence of axonal connections) and effective connectivity (the causal influences mediated by these connections). The deployment of anatomical connections provides important constraints on effective connectivity, but does not fully determine it, because synaptic connections can be expressed functionally in a dynamic and context-dependent fashion. Although it is generally assumed that anatomical connectivity data is important to guide the construction of neurobiologically realistic models of effective connectivity; the degree to which these models actually profit from anatomical constraints has not yet been formally investigated. Here, we use diffusion weighted imaging and probabilistic tractography to specify anatomically informed priors for dynamic causal models (DCMs) of fMRI data. We constructed 64 alternative DCMs, which embodied different mappings between the probability of an anatomical connection and the prior variance of the corresponding of effective connectivity, and fitted them to empirical fMRI data from 12 healthy subjects. Using Bayesian model selection, we show that the best model is one in which anatomical probability increases the prior variance of effective connectivity parameters in a nonlinear and monotonic (sigmoidal) fashion. This means that the higher the likelihood that a given connection exists anatomically, the larger one should set the prior variance of the corresponding coupling parameter; hence making it easier for the parameter to deviate from zero and represent a strong effective connection. To our knowledge, this study provides the first formal evidence that probabilistic knowledge of anatomical connectivity can improve models of functional integration.

## Introduction

One of the key themes in biology is the characterisation of structure–function relationships. For example, the range of functional interactions a protein can engage in depends on its three-dimensional structure. In the brain, a similar relationship exists between anatomical and effective connectivity. The former denotes the presence of axonal connections among neurons or neuronal populations and the latter refers to the causal influences that are mediated by these connections (Aertsen and Preißl, 1999; Friston, 1994). It is generally accepted that anatomical connectivity provides important constraints on effective connectivity. For example, it has been shown that the functional repertoire of a cortical area (the “functional fingerprint”) is closely related to the pattern of its anatomical connections (the “connectional fingerprint”) (Passingham et al., 2002). Similarly, network analyses of anatomical connectivity (Hilgetag et al., 2000) and functional interactions in the Macaque cortex (Stephan et al., 2000) have revealed similar clustering in the cortical network and indicated that, both from an anatomical and functional perspective, the Macaque cortex possesses small world properties. Finally, a number of analytical and simulation studies have recently started to explore how different types of structural network topologies are linked to different types of neuronal dynamics (Bullmore and Sporns, 2009; Honey et al., 2007; Sporns et al., 2002; Strogatz, 2001).

It is important to note that anatomical connectivity constrains but does not determine effective connectivity. There are several reasons for this. First, the function of a synapse depends on its recent history. For example, in the absence of any structural changes of the synapse *per se*, marked facilitation or depression of synaptic transmission can occur at a timescale of milliseconds (Zucker and Regehr, 2002). A second, and probably more important, reason why there is no one-to-one mapping between anatomical and effective connectivity is that the structural presence of a synaptic connection does not determine whether it will be engaged during a particular process or not. Various mechanisms exist by which synaptic connections can be enabled or disabled in a dynamic fashion at the timescale of milliseconds. These mechanisms include gating and gain control mechanisms, which render synaptic transmission dependent on the current membrane potential and the history of other synaptic inputs nearby (see Discussion). These transient and nonlinear effects are important for explaining the dynamics of functional interactions among neuronal populations (Friston et al., 1995; McIntosh, 2000; Salinas and Sejnowski, 2001; Stephan et al., 2008a).

**Fig. 1:**
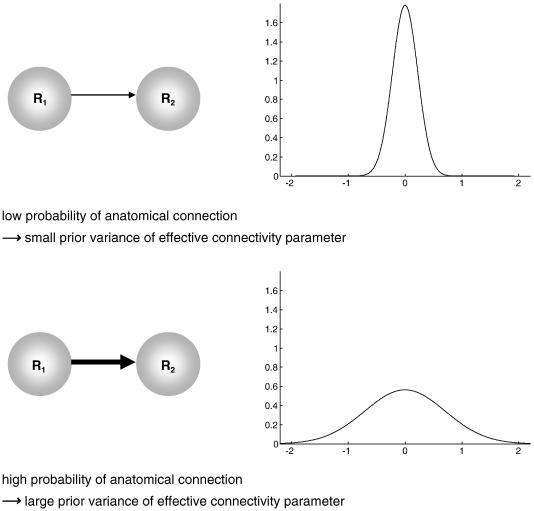
This figure provides a schematic summary of the intuitive notion that a higher probability that a connection between two regions R_1_ and R_2_ exists anatomically should be associated with a larger prior variance of the corresponding effective connectivity parameter in DCM, hence making it easier for the parameter to deviate from its prior mean of zero and therefore represent a strong (negative or positive) effective connection.

Major efforts have been made over the last decade to make anatomical connectivity data available for constructing better models of brain function, ranging from construction of large scale databases of anatomical tract tracing data (Bota et al., 2005; Burns and Young, 2000; Scannell et al., 1999; Stephan et al., 2001) to analyses of human brain connectivity by non-invasive diffusion weighted imaging (Alexander, 2005; Behrens et al., 2003; Jones et al., 1999; Kaden et al., 2008; Le Bihan, 2003; Mori and Zhang, 2006; Parker et al., 2002; Tuch et al., 2005). So far, however, no study has formally investigated to what degree models of effective connectivity profit from detailed quantitative knowledge of anatomical connectivity. Here, we present such an investigation by combining probabilistic tractography and dynamic causal models (DCMs) (Friston et al., 2003) of fMRI data. Our approach rests on a simple idea: we use an estimate of the probability that a given anatomical connection exists, as provided by tractography, to constrain the likely range of the effective connection strength. More formally, the anatomical likelihood of a given connection is used to inform the prior variance of the corresponding coupling parameter in the DCM. Such anatomically informed priors can have different forms. The most intuitive notion is that the higher the likelihood that a given connection exists anatomically, the larger one should set the prior variance of the corresponding effective connectivity, making it easier for the parameter to deviate from its prior mean of zero and therefore represent a strong (negative or positive) connection. This is shown schematically by Fig. 1.

In the present study, we constructed 64 alternative DCMs, each of which embodied a different mathematical mapping between the anatomical probability of a given connection and the prior variance of the corresponding coupling parameter. Some models embodied the (intuitive) notion that the prior variance should increase with anatomical likelihood; these models only differed with regard to the mathematical relationship between anatomical connection likelihood and prior variances in DCM. Other models represented the counterintuitive notion that the prior variance of coupling parameters in DCM should decrease with increasing anatomical likelihood. As a reference, we also used several naive models in which the prior variances of the coupling parameters in DCM were independent of the anatomical likelihood of the corresponding connections. Using Bayesian model selection (Penny et al., 2004; Stephan et al., 2007b), we tested which of these models best explained experimentally measured fMRI data in a DCM of four visual areas (Stephan et al., 2007a), using data from a group of twelve healthy volunteers. Our comparisons showed that the best model (i.e., the model with the highest evidence) is one in which anatomical probability increases the prior variance of DCM coupling parameters in a nonlinear and monotonic (sigmoidal) fashion. In contrast, models that ignore anatomical connectivity have substantially less evidence. To our knowledge, this study provides the first empirical evidence that probabilistic knowledge of anatomical connectivity can improve models of functional integration in the brain.

This paper comprises three sections. In the first we review DCM, with a special focus on the construction of anatomically informed priors on the coupling parameters. In the second section, we describe the data and how probabilistic tractography measures were derived. In the final section, we present the results of Bayesian model comparisons used to establish if and how anatomical constraints are evident in functional data.

## Theory

### Dynamic causal modelling (DCM)

DCM for fMRI uses a bilinear model of neural dynamics in a system of *n* distributed brain regions, where neural population activity in each region is represented by a single state variable. DCM models the change of this neural state vector *x* using the following differential equation:

(1) $$\frac{dx}{dt}=\left(A+\sum_{j=1}^{m}u_{j}B^{\left(j\right)}\right)x+Cu\text{.}$$

Here, the *A* matrix represents the endogenous (context-independent or fixed) strength of connections between the regions, and the matrices *B*^(*j*)^ represent the modulation of these connections (e.g. due to learning, attention, etc.) induced by the *j*th input *u*_*j*_. Finally, the *C* matrix represents the influence of direct (exogenous) inputs to the system (e.g. sensory stimuli). Note that all parameters are rate constants and are thus in units of s^− 1^.

To explain regional BOLD responses, DCM for fMRI combines this model of neural dynamics with a biophysically motivated hemodynamic model; for details see Friston et al. (2000) and Stephan et al. (2007b). Together, the neural and hemodynamic state equations furnish a deterministic forward model with hidden biophysical states. For any given combination of parameters *θ* and inputs *u*, the measured BOLD response *y* is modelled as the predicted BOLD signal *h*(*u*,*θ*) plus a linear mixture of confounds *Xβ* (*e.g*., signal drift) and Gaussian observation error *e*:

(2) y=h(u,θ)+Xβ+e.

DCM uses a fully Bayesian approach to parameter estimation, with empirical priors for the hemodynamic parameters and conservative shrinkage priors for the coupling parameters; see Friston (2002) and Friston et al. (2003) for details. Briefly, the posterior moments are updated iteratively using variational Bayes, under a fixed-form Laplace (i.e., Gaussian) approximation, *q*(*θ*), to the conditional density *p*(*θ *| *y*). This uses gradient ascent on a free-energy bound on the log-marginal likelihood, ln *p*(*y* | *m*), for a particular model, *m*. This optimises the maximum a posteriori (MAP) estimates of the parameters in the E-step of an EM algorithm, whereas the M-step optimises hyperparameters *λ* that control the covariance components of the observation error *e*.

**Fig. 2:**
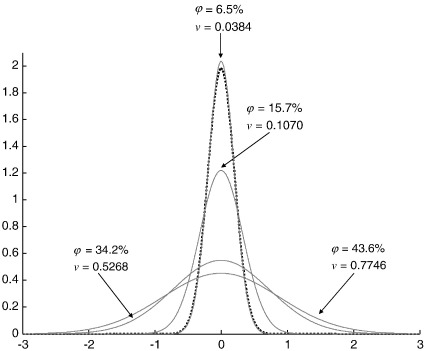
This figure shows different Gaussian priors for coupling parameters in the DCM shown by Fig. 3. The dotted black line represents the prior variance, *v* = 0.0405, that was originally suggested as a default value for a four-area DCM (Friston et al., 2003). The solid grey lines represent connection-specific prior variances that result from transforming the anatomical connection probabilities *φ*_*ij*_ (Fig. 3) using Eq. (6) and the hyperparameters of the optimal model *m*_45_ (with hyperparameters *α *= 4, *β *= 12; see Fig. 4).

Of particular interest are the Gaussian shrinkage priors *p*(*θ *| *m*) that constrain the estimates of the coupling matrices (i.e. the *A* and *B* matrices in Eq. (1)). They are called shrinkage priors because they have a prior mean of zero and a small prior variance (i.e. high prior precision) and thus shrink the posterior estimates of coupling parameters in DCM towards zero. The choice of shrinkage priors in DCM was motivated by two reasons: (i) they enforce conservative parameter estimation, and (ii) ensure the system is dissipative.^2^ These considerations led to a quantitative heuristic for the prior variance of coupling parameters, which depends on the number of areas in the system (Friston et al., 2003). For example, for a four-area model as in the present study, the prior variance of coupling parameters is set to 0.0405. Fig. 2 shows this default prior (bold dotted line) and contrasts it with anatomically informed priors (specifically, those which were found to be optimal by our model selection procedure described below).

Critically, the higher the prior variance of coupling parameters, the easier it is for posterior estimates to deviate, in either direction, from the prior mean of zero. In DCM, the shrinkage priors on the coupling parameters are identical for all connections in the system, regardless of whether they are anatomically likely or not. In the next section, we motivate a scheme by which anatomical measures from probabilistic tractography can be used to define the prior variance for individual connection strengths and thus constrain dynamic causal models anatomically.

### Anatomically informed priors

#### Anatomical measures

Imagine that we are given some (probabilistic) measure *φ*_*ij*_^⁎^ of anatomical connectivity between regions *i* and *j* in the DCM. Generally, these will describe a probability density; for example, the probability of a streamline from a seed region to reach a target region. Because these measures are densities their absolute values depend on their units of measurement (e.g., per voxel). We can resolve this dependency by working with the relative probabilities on *n* connections

(3) $$\varphi_{ij}=\frac{\varphi_{ij}^{*}}{\sum_{ij}\varphi_{ij}^{*}}\text{.}$$

**Fig. 3:**
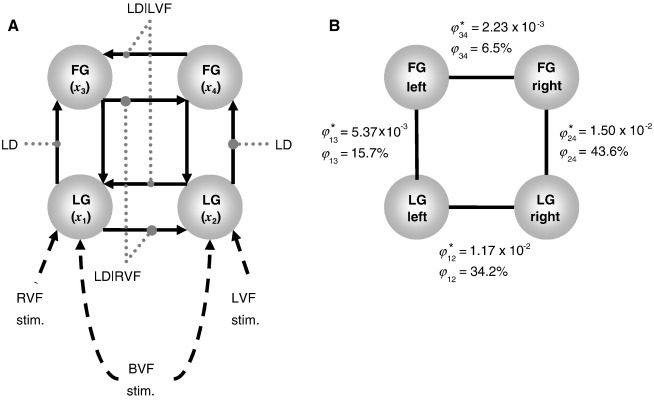
(A) Summary of the DCM used in this evaluation study; for details see (Stephan et al., 2007a). This four-area model included the reciprocally connected lingual gyrus (LG) and fusiform gyrus (FG) in both hemispheres (black solid lines). Non-foveal visual stimuli (words) were presented in either the right (RVF) or left (LVF) visual field with a randomized stimulus onset asynchrony between 1.5 and 2.5 s during 24 s blocks; these were modelled as individual events driving contralateral LG activity (black dashed lines). During the instruction periods, bilateral visual field (BVF) input was provided for 6 s; this was modelled as a box-car input to LG, in both hemispheres. Connections were modulated by task and stimulus properties (grey dotted lines). Intra-hemispheric LG → FG connections were allowed to vary during a letter decision (LD) task, regardless of visual field. In contrast, inter-hemispheric connections were modulated by task conditional on the visual field (LD|LVF and LD|RVF). (B) Mean anatomical connection probabilities at the group level. For each connection, its probability *φ*_*ij*_^⁎^ was computed using each participating region as a seed region once and then computing the average. This was done for each subject and the results were then averaged across all 42 subjects. For all connections the standard error across subjects was considerably smaller than the mean connection strength: *SE*(*φ*_12_) = 3.18 × 10^− 3^, *SE*(*φ*_13_) = 1.08 × 10^− 3^, *SE*(*φ*_24_) = 2.90 × 10^− 3^ and *SE*(*φ*_12_) = 7.68 × 10^− 4^. *φ*_*ij*_ represent normalised connection probabilities (c.f. Eq. (3)).

This ensures that ∑*φ*_*ij *_= 1 and furnishes a measure of anatomical strength for any one connection, relative to all others. In Fig. 3B, *φ*_*ij*_ and *φ*_*ij*_^⁎^ are shown alongside the connections of the DCM considered in this study. We now have to consider how this relative measure might enter a probabilistic model of effective connectivity like a DCM.

#### Anatomically informed priors

In DCM, priors on the effective connectivity *θ*_*ij*_ between regions *i* and *j* are Gaussian shrinkage priors (see above)

(4) $$p\left(\theta_{ij}\right)\propto \exp\left(\frac{1}{2}\prod_{ij}\theta_{ij}^{2}\right)$$

where Π_*ij*_ is the prior precision of the connection. When the precision is large, the connections are constrained to be small*; i.e*., they shrink to their prior expectation of zero. This form allows us to specify anatomically informed priors *p*(*θ*_*ij*_) → *p*(*θ_ij_ *| *φ*_*ij*_), which change monotonically as a function of the anatomical connection strength, by making the precisions a function of *φ*_*ij*_:

(5) $$\Pi_{ij}=\Pi_{0}+\exp\left(\alpha -\beta \varphi_{ij}\right)\text{.}$$

If *β *> 0, as the relative strength *φ*_*ij*_ increases, the precision will decrease toward a lower bound Π_0_. In other words, if the anatomical measure suggests that a given connection is more probable than other connections, then the prior precision will be small, relaxing the shrinkage on the posterior estimates which, consequently, can take large values in either positive or negative direction. The relative effects of anatomical constraints will be less and less pronounced as *β* approaches zero. At *β *= 0, the anatomical measures play no role, and we are dealing with “anatomically uninformed” priors. Here, the degree of shrinkage is the same for all connections and is determined entirely by exp(*α*) ≥ 0. Finally, for *β < *0, the prior precision increases with the anatomical likelihood of a given connection, resulting in counterintuitive priors that shrink the posterior estimates more strongly for probable connections than for improbable ones. In summary, one can regard the hyperparameters *α* and *β* as controlling generic and anatomical constraints, respectively. Because these hyperparameters are unknown, they have to be optimised with respect to the model evidence, as described below.

#### Prior variances

Usually, priors are specified not in terms of precisions but in terms of variances

∑*_ij_* = Π_*ij*_^− 1^. In the case of anatomically informed priors we have

(6) $$\Sigma_{ij}=\frac{1}{\Pi_{0}+\exp\left(\alpha -\beta \varphi_{ij}\right)}=\frac{\Sigma_{0}}{1+\Sigma_{0}\exp\left(\alpha -\beta \varphi_{ij}\right)}\text{.}$$

**Fig. 4:**
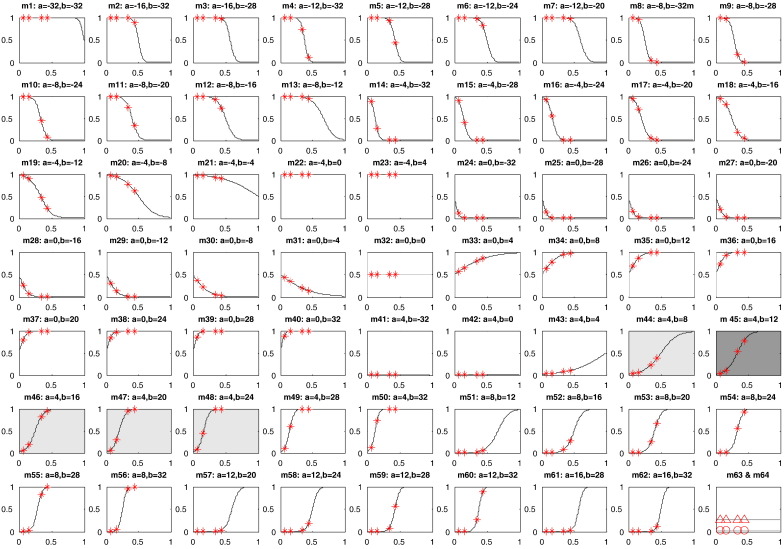
This figure summarises all 64 models tested by showing their mapping functions (specified by hyperparameters *α* and *β*; see Eq. (6)) that converted anatomical connection probabilities into prior variances of effective connectivity parameters in the DCM. Red stars represent the prior variances that result from transforming the anatomical connection probabilities *φ*_*ij*_ shown in Fig. 3. The plot in the right lower corner shows the prior variances for two models, *m*_63_ (red triangles) and *m*_64_ (red circles). The top 5 models, as established by Bayesian model selection (see Fig. 5 for details), are shown with a grey background. Note that these were the only models with a non-negligible posterior probability (larger than 10^− 4^; compare Fig. 5C). The best model, *m*_45_*,* is highlighted by a dark grey background; its prior variances for the effective connectivity parameters are shown in Fig. 2.

This is simply a logistic sigmoid function of *φ*_*ij*_. Here, the sigmoid is bounded by the upper limit on variance ∑_0_ (e.g., imposed by dynamical stability priors), where ∑_0_ is the inverse of Π_0_ in Eq. (5). The point of inflection (i.e., shift) is determined by *α*, while *β* controls the slope (i.e., gain) of the sigmoid. Most importantly, the prior variance; (i) increases monotonically with *φ*_*ij*_ if *β *> 0, (ii) is unaffected by anatomical information if *β *= 0, and (iii) decreases monotonically with *φ*_*ij*_ if *β *< 0. This perspective on anatomically informed priors p(*θ_ij_ *| *φ*_*ij*_) highlights its flexibility and form (see also Fig. 4).

### Are anatomically informed priors useful for DCM?

The critical issue is, of course, whether DCMs with anatomically informed priors are better than DCMs which discount anatomical connectivity. This can be addressed by comparing the model evidence of DCMs with different hyperparameters {*α*, *β*, *∑*_0_}, using Bayesian model selection (Penny et al., 2004; Stephan et al., 2007b). In particular, the value of *β* which optimises the model evidence indicates whether and how anatomically informed priors improve the model.

In this study, we compared different anatomically informed and uninformed priors using an established four-region DCM of visual responses measured during a paradigm requiring letter decisions and spatial decisions about identical word stimuli (Stephan et al., 2007a); here, we focus on the modulation of connectivity induced by letter decisions. The fMRI data came from a group of twelve healthy volunteers (all male and right-handed, mean age 24.9 years, SD 3.4).

Using this DCM, we fixed the upper bound hyperparameter ∑_0_ to unity and optimised the remaining hyperparameters with respect to the model evidence by searching over model space; where each model had different values of *α* and *β*. Specifically, both these hyperparameters were varied in steps of 4 over the range [− 32, 32], resulting in 289 different models. For each of these models, we computed the associated prior covariance matrix according to Eq. (6), based on anatomical connectivity estimates which are described in the next section and summarised by Fig. 3B. In a subsequent pruning step, we compared these matrices and removed those showing only small differences (i.e., where the maximal difference between corresponding elements in the covariance matrices was less than 10^− 2^). This resulted in 62 sets of hyperparameters and associated models, which will be referred to as *m*_1_⋯*m*_62_. These DCMs were distinguished only by different anatomically informed (*β *≠ 0) or anatomically uninformed (*β *= 0) priors. As additional controls, we considered two further models with anatomically uninformed priors: one model, *m*_63_, used the default shrinkage priors in DCM (i.e. a prior variance of 0.0405 for each connection; see red circles in Fig. 4). The second, *m*_64_ was constructed by assuming that each connection was equally likely (i.e., *φ*_*ij *_= 25%), resulting in the same prior covariance for each connection (i.e. 0.2689; see red triangles in Fig. 4). All 64 models were fitted to the fMRI data from each of the 12 subjects and subsequently evaluated using Bayesian model selection (BMS), as described next.

### Bayesian model selection (BMS)

A decision about which of several competing models is optimal cannot be based only on the relative fit to the data (i.e., accuracy) but also needs to take into account differences in model complexity (i.e., the number of free parameters and the functional form of the generative model, Pitt and Myung, 2002). Penalizing for model complexity is important because while accuracy increases monotonically with complexity, at some point the model will start fitting noise that is specific to the particular data (i.e., “over-fitting”). Therefore, models that are too complex show poor generalisability. Given that all models are equally likely *a priori*, the question “which is the optimal model?” can be reformulated as “which model represents the best balance between fit and complexity?” This is the model that maximizes the marginal likelihood or model evidence:

(7) p(y|m)=∫p(y|θ,m)p(θ|m)dθ.

Here, the integration subsumes the number and conditional dependencies among model parameters *θ* (Friston et al., 2007; Penny et al., 2004). In this study, we approximate the log-evidence with the same free-energy bound, *F*, that is used to optimise the parameters above (Friston et al., 2007; Mac Kay, 2003; Neal and Hinton, 1998). As detailed elsewhere (Stephan et al., 2009), the advantage of *F* over other approximations (like the Akaike or Bayesian Information Criteria), is that it accounts properly for conditional dependencies among the parameter estimates. The derivation of *F* and its mathematical interpretation with regard to model accuracy and complexity have been described in detail in previous publications (Friston et al., 2007; Penny et al., 2004; Stephan et al., 2007b).

**Fig. 5:**
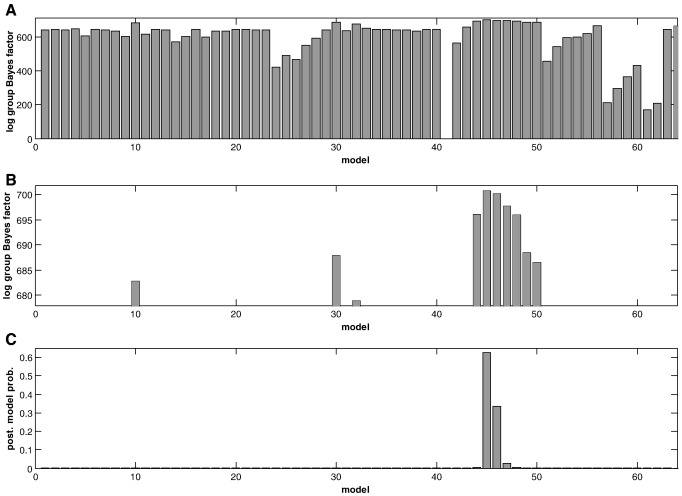
The log-evidence (pooled over subjects) for all 64 models, in relation to the worst model (*m*_41_), are shown in subplot A. Because the scaling makes it difficult to recognize the best model at a glance, the graph is redrawn in subplot B and thresholded such that only the top 10 models are plotted. It can now be seen easily that the best model is model *m*_45_*.* Furthermore, comparing this result with Fig. 4, it becomes apparent that 7 out of the 10 best models possess anatomically informed priors that accord with the intuitive notion that prior variance should increase with the probability or strength of an anatomical connection. The posterior probabilities of all models are shown by subplot C.

To quantify the relative evidence for two models *m*_*i*_ and *m*_*j*_ at the group level, we report differences in their log-evidence summed across subjects (c.f. Fig. 5); this is equivalent to using log group Bayes factors (Stephan et al., 2007b)

(8) $$\ln\prod_{n}BF_{ij}^{\left(n\right)}=\ln\prod_{n}\frac{p\left(y_{n}|m_{i}\right)}{p\left(y_{n}|m_{j}\right)}\approx \sum_{n}F_{i}^{\left(n\right)}-F_{j}^{\left(n\right)}$$

where *n* is an index over subjects. A difference in log-evidence (free-energy) of three or more is generally considered to be strong evidence for one model over another (Kass and Raftery, 1995). It is worth mentioning that although random-effects procedures for group-level BMS are available (Stephan et al., 2009), the fixed-effects BMS implicit in Eq. (8) is more appropriate here. This is because we are characterising a basic relationship between anatomical and effective connectivity that is fixed over subjects.

When each model is equally likely *a priori* (i.e. flat priors on models) the posterior probability of each model is proportional to the model evidence: *p*(*m_i_ *| *y*)∝*p*(*y* | *m*_*i*_). This means one can normalise the evidence for each model by dividing by the sum of evidences across models to give its posterior probability. The posterior probabilities for all models tested are shown in Fig. 5C.

## Methods

### Probabilistic tractography

#### Subjects

42 healthy volunteers (21 male) took part in the diffusion data acquisition, which was carried out at the Max Planck Institute for Human Cognitive and Brain Sciences at Leipzig, Germany. Subjects were on average 26.5 years old (range: 22–34; standard deviation: 2.8 years) and no subject had a history of neurological, psychiatric, or other major medical disorder. The study was approved by the local ethics committee of the University of Leipzig (Leipzig, Germany), and participants gave written informed consent. Data were handled anonymously.

#### Data acquisition and pre-processing

Diffusion weighted data and high-resolution three-dimensional T1 and T2 weighted images were acquired on a Siemens 3 T Trio scanner with an 8-channel array head coil and maximum gradient strength of 40 mT/m. The diffusion weighted data were acquired using twice-refocused spin-echo echo planar imaging (Reese et al., 2003) (TR = 12 s, TE = 100 ms, 72 axial slices, resolution 1.72 × 1.72 × 1.7 mm). We used a GRAPPA technique (with a reduction factor of 2.0) for parallel imaging. Diffusion weighting was isotropically distributed along 60 directions (Jones et al., 1999) with a *b*-value of 1000 s/mm^2^. The high angular resolution of the diffusion weighting directions improves the robustness of probability density estimation by increasing the signal-to-noise ratio and reducing directional bias. Additionally, seven data sets with no diffusion weighting (b0) were acquired initially and interleaved after each block of 10 diffusion weighted images as anatomical reference for motion correction. To further increase signal-to-noise, we acquired three consecutive scans, which were subsequently averaged together. The entire data acquisition protocol lasted approximately 45 min. Motion correction for the diffusion weighted images was applied to all images using 7-parameter global re-scale registration (Jenkinson et al., 2002) as implemented in the FSL software (FMRIB Software Library, University of Oxford, http://www.fmrib.ox.ac.uk/fsl). All baseline b0 images were aligned to a reference b0 image and the resulting linear transformation matrices were then applied to the diffusion weighted images following each baseline b0 image. The gradient direction for each volume was corrected using the rotation parameters. Then, the three scan repetitions were averaged to improve the signal-to-noise ratio.

#### Tractography

We applied the tractography approach as described by Kaden et al. (2007, 2008). This approach, which is based on the local fibre orientation density, computed by spherical deconvolution of the diffusion weighted signal, yields an estimate of the spatial probability distribution of connectivity from given seed regions. This approach is particularly useful for studies interested in anatomical connectivity between larger brain regions, which may not be adequately represented by single voxels (Koch et al., 2002) or single points (e.g. the centre of voxels; Behrens et al., 2003; Parker and Alexander, 2003). Instead, the concept of Kaden et al. extends the idea of connectivity to arbitrarily defined areas or volumes and defines anatomical connectivity as the proportion of fibre pathways originating in a specific source region that intersect a target region (Kaden et al., 2007). If the area or volume of the source region approaches a point, this measure reduces to the existence formulation proposed by Behrens et al. (Behrens et al., 2003), which only takes values on the discrete subset {0, 1}. It should be noted that none of the available tractography approaches makes it possible to determine the directionality of synaptic transmission along a given fibre tract; this is a general limitation of any connectivity metric based on diffusion weighted MRI.

#### Seed regions

Tractography was performed for each subject individually in his/her native (non-normalised) space. The resulting connectivity maps were then warped into a standard space (using the MNI 1 mm isotropic brain as a reference) for cross-subject averaging and comparison. To create seed masks for each subject, MNI coordinates were normalised to each subject's native space, using the inverse of the normalisation parameters. All resulting images were visually inspected to ensure that normalisation was successful and that each image was acceptable for analysis (e.g., in the correct orientation and not distorted).

To ensure that the computed tractograms were dominated by long-range connections, seed points were placed at the grey matter/white matter interface (white matter: fractional anisotropy; FA > 0.1). As the regional coordinates of fMRI time series used for the DCMs were located at the cortical surface, these coordinates were projected to the grey/white matter interface following the shortest (geodesic) path. Subsequently a seed region was defined by all points on the white matter surface within a radial distance of 3 voxels from the projected coordinates.

#### Connectivity measure

Considering all fibres originating in a given source region *S*, its structural connectivity with a given target region *T* can be defined in terms of the proportion of those fibres that intersect *T* while running within the brain white matter, yielding a number in the interval between zero (no fibres intercept *T*) and one (all fibres starting in *S* reach *T*) (Kaden et al., 2007). This quantity gives no information about the absolute number of connections between two regions, but reflects the degree of connectedness or relative connection density. It can be considered as a measure of the likelihood of a connection in the sense that it can be interpreted as the frequency at which one would reach *T* by randomly seeding a fibre starting within *S*. In our framework, the notions of anatomical connection strength and anatomical connection likelihood are therefore interchangeable.

One should note, however, that this connectivity metric may differ depending on whether *S* or *T* is chosen as the source region for fibre tracking (noting that this does not reflect the directionality of synaptic transmission along the pathways). For any given connection, one can remove this dependency on the seed region by computing the connectivity metric using each region as a seed region once and then averaging the result. Following this procedure, anatomical connectivity was estimated in each subject for all of the connections in the DCM (Stephan et al., 2007a) above and shown in Fig. 3. The subject-specific anatomical connection probabilities were then averaged across subjects; the resulting group values are reported alongside the corresponding connections in Fig. 3B. Note that the anatomical connection probabilities *φ*_*ij*_^⁎^ resulting from our procedure are fairly small, as each region is most likely connected to many more brain areas than those included in the DCM. Fig. 3B also shows the normalised connection probabilities *φ*_*ij*_ for each connection (c.f. Eq. (3)).

Our numerical implementation for computing the above connectivity measures was identical to that described in Kaden et al. (2007). Streamline tractography was run from 100 randomly sampled starting points per voxel in the seed region; i.e. about 2400 times on average. A distribution of the connectivity value was then obtained by repeating this procedure 1000 times with different realizations of the local model, sampled using the Metropolis–Hastings algorithm. This means about 2.4 million fibres were computed for each connectivity value.

## Results

Fig. 4 shows the relationship between the anatomical connection probability and prior variance of coupling parameters in DCM for all 64 models tested (note that the anatomically uninformed models *m*_63_ and *m*_64_ are shown in the same subplot). The log-evidences for all 64 models, in relation to the worst model (*m*_41_) are shown in Figs. 5A and B. Fig. 5C shows the corresponding posterior probabilities for all models. It can be seen that the best model is model *m*_45_. In this model, the DCM priors are anatomically informed, such that the prior variance of a coupling parameter in DCM increases monotonically with the anatomical likelihood of the connection. This result is pleasing because it confirms the intuitive notion that (i) probabilistic knowledge of anatomical connectivity should inform coupling strengths in dynamic models, and, more specifically, (ii) the more likely the anatomical connection, the easier it should be for the corresponding coupling to be expressed functionally and take large values.

Comparing Fig. 5B with Fig. 4, it is apparent that the next 6 best models, *m*_44_ and *m*_46_–*m*_50_, use anatomically informed priors with a similar form to the optimal model, i.e. priors that relax shrinkage when the strength of an anatomical connection increases. It is also worth noting that only the five best models, *m*_44_–*m*_48_, show a non-negligible posterior probability (i.e. larger than 10^− 4^; see Fig. 5C). These models are highlighted with a grey background in Fig. 4.

Two of the top 10 models, *m*_10_ and *m*_30_, possessed counterintuitive priors (where anatomical likelihood decreased prior variance). When comparing model *m*_45_ to model *m*_30_, the best counterintuitive model, the difference in log-evidence was 12.89 in favour of *m*_45_. This means that the group fMRI data are exp(12.89) ≈ 4 × 10^5^ times more likely under model *m*_45_ than under model *m*_30_.

Several of the models tested had anatomically uninformed priors, with the same prior variance for all coupling parameters, regardless of the anatomical likelihood of the respective connection. These models either (i) resulted from our systematic search over model space (for *β *= 0 or for large values of *α* that shifted the sigmoid such that all probabilities came to lie on the asymptote), (ii) were included as an additional control case (*m*_63_, assuming that each connection was equally likely, i.e. *φ*_*ij *_= 25%), or (iii) used the default prior variances as originally defined for DCM (*m*_64_). Notably, the corresponding uninformed prior covariances varied considerably across models, ranging from very high (e.g. 1 in *m*_1_) to very low (e.g. 0.018 in *m*_42_). As shown by Fig. 5, *m*_45_ performed considerably better than all models with uninformed priors, regardless of whether these used coupling parameters with high, intermediate or low prior variances. Specifically, when comparing model *m*_45_ to model *m*_32_ (the best of all models with anatomically uninformed priors), the relative log-evidence was 21.83 in favour of *m*_45_. This difference means that the observed group fMRI data are exp(21.83) ≈ 3 × 10^9^ times more likely under model *m*_45_ than under model *m*_32_.

## Discussion

In this study, we used probabilistic tractography based on diffusion weighted imaging data to obtain a measure of the anatomical likelihood of connections among visual areas. We then instilled these measures into an established dynamic causal model of interacting visual areas by making the prior variance of the DCM coupling parameters a function of the anatomical likelihood. A series of competing dynamic causal models was constructed and compared, using Bayesian model selection, to identify the most likely mapping between anatomical estimates of connectivity and prior variances in the DCM. In particular, we compared DCMs with anatomically informed and anatomically uninformed priors. Our results showed that the best DCM used anatomically informed priors where the prior variances of coupling parameters increased as a monotonic nonlinear function of anatomical likelihood.

This study is novel in two ways. To our knowledge, this is the first formal demonstration that knowing anatomical connectivity improves inference about effective connectivity. Although we cannot generalise to other data or paradigms, these results provide a sufficiency proof that anatomical information is useful in the context of modelling functional integration. Secondly, this study shows how anatomical and functional data can be integrated and analysed within the same inference framework for dynamic systems. In this context, we have demonstrated a generic model selection procedure that allows one to quantify the evidence for anatomical constraints in other settings. In the following, we discuss these two issues in detail.

Decades of anatomical and physiological work have shown that anatomical connectivity provides critical constraints on effective connectivity. In particular, the construction of large scale databases of neuroanatomical data over the last decade have made it possible to establish global relations between anatomical connectivity and brain function (Bota et al., 2005; Burns and Young, 2000; Hilgetag et al., 1996, 2000; Honey et al., 2007; Kötter and Stephan, 2003; Kötter et al., 2001; Passingham et al., 2002; Scannell et al., 1999; Sporns et al., 2000; Stephan et al., 2001; Young, 1992). However, it is equally clear that effective connectivity is only constrained, and not fully determined, by anatomical connections. There are numerous reasons for this, which are largely related to short-term synaptic plasticity and neuromodulation. For example, synapses can alter their transmission properties depending on the recent history of presynaptic and postsynaptic events. These phenomena include synaptic facilitation and depression and a variety of NMDA receptor-dependent mechanisms that change postsynaptic responsiveness at very short timescales, ranging from milliseconds to minutes (Bagal et al., 2005; Citri and Malenka, 2008; Montgomery and Madison, 2004; Passafaro et al., 2001; Zucker and Regehr, 2002). Short-term synaptic plasticity also has structural correlates at the level of synaptic proteins (which may be altered by very fast processes like phosphorylation), the number of membrane-bound NMDA and AMPA receptors, and molecular changes in intracellular signal transduction cascades. However, these structural changes live at a much smaller spatial scale than axonal connections *per se*. In other words, anatomical connectivity is usually described at a cellular scale, whereas neuronal communication and synaptic efficacy lie at the subcellular and molecular scale. An example of changes in synaptic efficacy is the phenomenon that a synaptic connection can dramatically alter its strength due to nonlinear dendritic integration of multiple synaptic inputs. For example, in the presence of non-inactivating dendritic sodium conductances (Schwindt and Crill, 1995) or dendritic calcium conductances activated by back-propagating action potentials (Larkum et al., 2004) the postsynaptic response at a given dendritic synapse can depend strongly on the temporal and spatial distribution of other synaptic inputs. The fact that synaptic efficacy and effective connectivity are highly context-dependent has been described numerous times in the context of gain control (Salinas and Sejnowski, 2001), theories of neural context (McIntosh, 2000), functional integration (Friston, 2002b) and nonlinear gating of connections among neuronal populations (Stephan et al., 2008a).

For these reasons and others, it has been clear for a long time that even a perfect knowledge of the anatomical layout of brain connections would not enable us to predict the brain's functional integration or context-dependent dynamics (see also Ghosh et al., 2008). It seems equally clear, however, that although anatomical connectivity does not predict effective connectivity, it might provide an important constraint (Stephan et al., 2008b). Driven by this notion, a variety of studies have recently started to use empirical data sets of anatomical connectivity data as a basis for large scale simulations of brain function (Honey et al., 2007; Jirsa, 2004; Sporns et al., 2000; Tagamets and Horwitz, 1998) or have investigated what types of network structures give rise to certain types of neuronal dynamics (Strogatz, 2001). Also, since their first application (McIntosh and Gonzalez-Lima, 1991), models for inferring effective connectivity from neuroimaging data have exploited existing knowledge of anatomical connectivity to specify model structure. This assumed that model quality should be improved by incorporating anatomical knowledge. While this notion is plausible, it has not been tested formally. The present study has provided evidence in favour of this assumption.

A variety of attempts have been made over the last few years to use estimates of anatomical connectivity as derived from diffusion weighted imaging, to constrain or refine analyses of fMRI data. For example, some studies have shown that specific brain areas can be delineated on the basis of their anatomical connectivity pattern (Anwander et al., 2007; Tomassini et al., 2007), and that this delineation is well matched by fMRI activity in tasks that activate the area in question (Behrens et al., 2006; Friederici et al., 2006; Johansen-Berg et al., 2004, 2005; Klein et al., 2007). Some work has also addressed joint analyses of anatomical and functional connectivity. For example, Koch et al. (2002) correlated the results from functional connectivity analyses of fMRI data and tractography based on diffusion weighted imaging data. They found that the relation between anatomical and functional connectivity did not follow a simple rule but varied considerably across regions. More recently, it has been suggested that anatomical and functional connectivity, as inferred from fMRI data, could be analysed within a single mathematical framework (Jbabdi et al., 2007). Our study extends these efforts by linking anatomical connectivity estimates to inferences about effective connectivity. The proposed use of anatomically informed priors in dynamic causal models represents a simple approach that may prove useful in other contexts.

Clearly, there are many ways that anatomically informed priors in DCMs could be formulated mathematically. Nevertheless, even in this initial study, we investigated a relatively large range of different models, using a generic formulation that was able to model a variety of different relationships between the anatomical likelihood of a connection and the prior variance of the associated DCM coupling parameter. Notably, this set of 64 models included DCMs in which the prior variance increased with anatomical likelihood (as one would expect intuitively), DCMs in which the prior variance decreased with anatomical likelihood (i.e. counterintuitive models), and models in which the anatomical probability did not inform the priors (i.e., all connections were treated equally). All models were then compared using Bayesian model selection, a technique that takes into account not only the fit, but also the relative complexity of competing models. Differences in model complexity arise not only by inclusion of additional parameters, but also through differences in their prior variance. Simply speaking, increasing the prior variance of parameters endows a model with more effective degrees of freedom to fit the data and therefore renders it more complex (formally speaking, model complexity increases with the Kullback-Leibler divergence between the posterior density and the prior density; for details, see Stephan et al., 2009).

Figs. 4 and 5 summarise the results of our model comparison procedure. It is striking that the seven best models, *m*_44_–*m*_50_, use anatomically informed priors of an intuitive sort, i.e. priors that relax shrinkage when the strength of an anatomical connection increases. One may wonder why these particular anatomically informed priors fared better than formally similar priors. One explanation may be that optimal models tended to preclude very low prior variances, whereas in other models with priors of a similar form the less likely connections (i.e. left LG ↔ left FG and left FG ↔ right FG) had very low prior variances (compare Fig. 4). Low prior variances have opposing effects on model evidence (c.f. the appendix to Stephan et al., 2009): on one hand, as mentioned above, low prior variance increases model evidence by reducing the effective degrees of freedom and thus model complexity. On the other hand, because any difference between the posterior and prior means also contributes to model complexity, low prior variance means that any deviation of posterior estimates from zero will incur a higher cost (higher model complexity) than in models with high prior variance. These opposing effects on model complexity prohibit trivial strategies for choosing priors, such as generally minimising or maximising prior variance for all connections, and illustrate the necessity of establishing procedures for defining connection-specific prior variances, as suggested in this paper. In the present study, our BMS results imply that explaining the observed data required that connections with comparatively small anatomical likelihood nevertheless possessed non-negligible effective strengths.

After the seven best models, the next-best three models exhibited either anatomically informed but counterintuitive priors (in which anatomical likelihood decreased the prior variance) or anatomically uninformed priors, with the same prior variance for all coupling parameters, regardless of the anatomical likelihood of the respective connection (Fig. 5). Why these particular models were better than some models with anatomically informed and intuitive priors is not clear. However, on the whole, the relative evidence for these models was very low. They were considerably worse than the best model (with group Bayes factors of 10^5^–10^9^ in favour of the latter). Additionally, without exception, their posterior probabilities were very close to zero (i.e. below 10^− 4^; see Fig. 5C), while the posterior probability of the best model was 63%.

The present study has a number of limitations. First, the functional and structural data were not obtained from the same subjects. The diffusion weighted MRI data were acquired from a group of 42 healthy volunteers, and the tractography values used in this study represent averages across this population. Anatomical connection probabilities were estimated for regions defined by fMRI activations in a second population of 12 subjects (Stephan et al., 2007a); these functional data were used to fit the DCMs. It is possible that our model selection approach might have given different hyperparameter estimates if we had used structural and functional data from the same subjects. We do not think a potential difference is likely to be substantial, because we averaged the tractography results across a relatively large group of 42 volunteers, thus diminishing any impact of inter-individual variation. We will revisit this issue in future studies that obtain structural and functional data from the same subjects. Concerning the present study, we would like to stress that its purpose was not to obtain exact hyperparameter estimates. We wanted to see whether tractography-informed priors improved the model evidence; and if so, what form the mapping from anatomical probability to prior constraint might take.

A second limitation, as in all modelling studies, is that only a limited number of models could be tested. We addressed this issue by defining a relatively large model space (defined by two parameters in the sigmoidal mapping) and systematically searching this model space. A third limitation, shared by all studies that rely on diffusion weighted imaging, is that our estimates of anatomical connectivity are non-directional. That is, having established (probabilistically) that there is a connection between region *S* and region *T*, we do not know whether the direction of synaptic transmission is from *S* to *T*, from *T* to *S*, or in both ways. In our study, this constraint is only relevant in that the prior variances for a pair of reciprocal connections are the same; in contrast, our DCMs allow for separate estimates of effective connectivity in the two directions.

Third, a general problem of many probabilistic tractography procedures, as with the one used in this study, is that the measure of connectivity between two regions is influenced by their spatial distance. This is due to the accumulation of errors over space when deriving connectivity measures from sequential tractography. In the network examined in this study, the distances traversed by intra-hemispheric connections are shorter than for inter-hemispheric connections. It is thus possible that the anatomical connectivity measures for the latter are underestimated relative to the former. However, for highly coherent fibre bundles, like the corpus callosum, this effect may not be critical: although no formal analysis exists, this was demonstrated anecdotally in previous work using the same tractography method (c.f. Figs. 7 and 8 in Kaden et al., 2007). This issue is a potential confound that could be addressed in future studies by including a distance hyperparameter in the prior covariance model and evaluating its contribution using Bayesian model comparison as above.

Finally, it should be noted that our proposed method is only useful when dealing with a network of more than two regions. This is because of the non-directional nature of diffusion data (see above) and because we are dealing with relative probabilities; where *φ*_*ij *_= 100% for a two-area network (c.f. Eq. (3)). This is not a severe limitation because most applications of DCM concern networks with more than two areas.

Our approach could be extended to overcome some of the limitations mentioned above. In this paper, we optimised hyperparameters controlling the way tractography measures provide prior constraints on effective connectivity. This optimisation was with respect to a free-energy bound on the ensuing log model evidence, using a systematic search of hyperparameter space. In principle, this optimisation could be part of a hierarchically extended model that included the hyperparameters as unknown quantities. This would entail formulating priors on these hyperparameters (i.e., a prior on a prior or hyperprior) and extending the model inversion scheme to accommodate covariance functions that are nonlinear in the hyperparameters. Then, the explicit search over hyperparameters used in this paper could be replaced by optimisation of a single model. An additional advantage of this procedure would be that we would have a posterior density on the hyperparameters themselves, allowing us to quantify how certain one was about the contribution of anatomical constraints. As noted by one of our reviewers, another advantage of such hierarchically extended models is that they would allow for different priors on the two directions of a reciprocal connection. The latter is not possible within the present framework because the non-directional nature of tractography data enforces the same anatomical connection probability for both directions, giving identical prior variances. However, if we used the anatomical connection probability to specify a hyperprior, the prior variances could change adaptively, in a direction-specific fashion. This would account, for example, for possible asymmetries in forward and backward connections.

In summary, using a combination of probabilistic tractography, dynamic causal modelling and Bayesian model selection, we have demonstrated that probabilistic estimates of anatomical connection strengths can be used to improve models of effective connectivity. We expect that this type of approach will prove useful in future studies of functional integration that have access to both fMRI and diffusion weighted imaging data.

## Footnotes

2 In DCM as implemented in SPM5/SPM8, stability of the system is guaranteed by using lognormal priors on the negative self-connections; see the discussion on stability in Stephan et al. (2008).
